# Comparative viral inactivation kinetics of electron beam and gamma irradiation and functional characterization of electron beam inactivated viruses

**DOI:** 10.3389/fmicb.2026.1963124

**Published:** 2026-10-20

**Authors:** Muzaffar Ali, Chandni Praveen, Suresh D. Pillai

**Affiliations:** 1 National Center for Electron Beam Research, Texas A&M University, College Station, TX, United States; 2 Department of Food Science and Technology, Texas A&M University, College Station, TX, United States

**Keywords:** antigen preservation, electron beam, gamma radiation, human rotavirus, influenza A virus, respiratory syncytial virus, virus host interaction, virus inactivation

## Abstract

**Introduction:**

Electron beam (eBeam) technology is increasingly being adopted in biomedical research and industry as an alternative to cobalt-60-based gamma irradiation. However, a comprehensive understanding of how eBeam energy and dose influence viral inactivation and the preservation of viral surface antigens remains limited. This study compared the inactivation kinetics of human rotavirus (RV), human respiratory syncytial virus (RSV), and influenza A virus (IAV) following exposure to high-energy electron beam (HEEB; 10 MeV), medium-energy electron beam (MEEB; 4.5 MeV), and gamma irradiation under conditions that were standardized to the greatest extent possible. Additionally, antigenic characterization was performed for HEEB and MEEB inactivated viruses.

**Methods:**

Human rotavirus, human respiratory syncytial virus, and influenza A virus were irradiated using HEEB, MEEB and gamma irradiation using incremental doses under standardized experimental conditions. Viral inactivation kinetics (D_10_) were determined using TCID_50_ assays while preservation of viral antigenicity and virus–host receptor interactions were evaluated by hemagglutination and ELISA assays. Heat and formalin inactivation were used as experimental controls.

**Results:**

All three modalities effectively inactivated the tested viruses with comparable overall inactivation efficiency. The D_10_ values of RV (1.23–1.29 kGy) and RSV (1.03–1.73 kGy) did not differ significantly (*p* = 0.01) among the irradiation modalities. The IAV exhibited greater radioresistance, with D_10_ value ranging between 1.62–2.27 kGy. Complete inactivation by both high- and medium-energy eBeam was confirmed by three serial passages. Importantly, eBeam-treated viruses retained viral antigenicity (VP4, F-protein, and HA) at levels comparable to untreated controls, whereas heat and formalin treatments reduced antigenicity by 5.09- to 256-fold and 1.26- to 2-fold, respectively. Furthermore, eBeam-inactivated viruses exhibited 1.54- to 23.11-fold greater virus-host receptor recognition than heat and formalin inactivated preparations.

**Discussion:**

Both high-energy and medium-energy electron beam modalities achieved viral inactivation efficiencies comparable to gamma irradiation under standardized conditions, supporting their potential as non-radioisotopic alternatives for viral inactivation. Importantly, eBeam treatment preserved viral antigenicity and functional virus–host receptor interactions more effectively than conventional heat and formalin inactivation methods. These findings highlight the potential of eBeam technology as a platform for producing inactivated viral preparations for vaccine development and other biopharmaceutical applications.

## 1 Introduction

Ionizing radiation technologies are extensively employed across medical, industrial, agricultural, environmental and biotechnological sectors owing to their ability to achieve effective microbial inactivation (Bhatia and Pillai, 2022; Skrobarczyk et al., 2022). The high penetration ability enables pathogen elimination within sealed packages and complex matrices, making them ideal for medical device sterilization, decontamination of biologics and vaccine development (Suljovrujic et al., 2024). Conventional inactivation strategies including thermal-inactivation (heat, steam), and chemical inactivation (formalin, beta-propiolactone, ethylene oxide gas) require longer incubation time and often damage structural proteins essential for immunogenicity (Delgado et al., 2009; Skrobarczyk et al., 2022). Chemical inactivation with formaldehyde introduces covalent crosslinks between amino acid residues, altering antigenic epitopes, while thermal inactivation induces global protein denaturation through disruption of non-covalent interactions maintaining tertiary structure (Murphy and Walsh, 1988; Butreddy et al., 2021). In contrast, ionizing radiation preferentially targets nucleic acids over structural proteins, achieving complete pathogen inactivation while preserving the native structural antigen conformation. This serves as a critical attribute for developing effective vaccines and immunomodulators (Buxton et al., 1988; Jonah et al., 1990; Fertey et al., 2020; Bhatia and Pillai, 2022; Perera et al., 2025; Praveen et al., 2025).

Among ionizing radiation modalities, gamma and electron beam (eBeam) represent the two principal technologies employed for sterilization and pathogen inactivation. Gamma radiation is generated by the decay of radioactive isotopes primarily cobalt-60 (^60^Co) or Cesium-137 (^137^Cs). Cobalt-60 emits gamma photons of two different energies of 1.17 MeV and 1.33 MeV (million electron volts) and emits low energy beta rays (electrons) with a maximum energy of 0.32 MeV (Cleland, 2006; Munir and Federighi, 2020). Electron beam is generated using machine-based electron accelerators such as linear accelerator (LINACs) or Rhodotrons operating using commercial electricity without any radioactive isotopes. Both gamma and eBeam inactivates pathogen through direct DNA/RNA damage with energetic electrons and via indirect damage to nucleic acids mediated primarily by the radiolytic products such as hydroxyl radicals (•OH), hydrogen peroxide (H_2_O_2_), aqueous electrons (e^−aq^), and hydrogen atom (•H) (Buxton et al., 1988; Jonah et al., 1990). While the underlying radiochemistry is shared, these modalities differ fundamentally in dose rate, particle energy, and penetration characteristics. Gamma photons exhibit deep penetration capability depending upon the product geometry and configuration making this modality a historical benchmark for sterilization of dense and heterogenous products in sealed final packaging (ISO 11137-2, 2013). However, gamma processing faces inherent constraints arising from the physics of radioactive decay. The delivered dose is governed by source activity, source-to product distance, facility design and source configuration, resulting in relatively low dose rates, typically ranging from 0.03 to 12 Gy/s (Tallentire et al., 2010). These low dose rates necessitate prolonged exposure time, often hours to achieve higher doses. Conversely, eBeam systems deliver equivalent doses at rates of 10^2^–10^4^ Gy/s, achieving complete inactivation within seconds. Furthermore, concerns related to radioactive source security, regulatory compliance, source replenishment, and long-term sustainability have increasingly challenged the widespread adoption of gamma irradiation technology (Bhatia and Pillai, 2022; Praveen et al., 2025).

Electron beam technology is available in multiple energy configurations, suited for specific applications based on penetration ability and product geometry. High Energy Electron Beam (HEEB) operates at 5–10 MeV and provide higher penetration compared to Medium Energy Electron Beam (MEEB) that operates at 1–5 MeV energy and widely used for food irradiation and medical device sterilization purposes. Comparatively, Low Energy Electron Beam (LEEB) (<1 MeV, typically 80–300 keV) achieves mostly surface level penetration and used for surface decontamination and material modification applications. All eBeam modalities have shown to effectively inactivate microbial pathogens under specific treatment parameters and material configurations suitable to uniformly deliver doses across the samples (Predmore et al., 2015; Fertey et al., 2020; Skrobarczyk et al., 2022). Electron beam modalities have significant operational advantages over gamma such as precise dose control, instant switch on/ off capability, elimination of radioactive isotope use, positioning eBeam as a sustainable alternative to gamma.

An effective viral inactivation method must ensure complete and irreversible loss of viral infectivity (Bonnafous et al., 2014). While chemical inactivation with formaldehyde or β-propiolactone achieves this goal, it has been shown to alter essential antigenic proteins in respiratory syncytial virus (Delgado et al., 2009), influenza A virus (Bonnafous et al., 2014), Japanese encephalitis virus (Fan et al., 2015), and poliovirus (Wilton et al., 2014). Ionizing radiation offers a distinct advantage by achieving complete inactivation while preserving antigenic surface proteins. This feature has been studied using gamma irradiation for influenza H3N2 (Salehi et al., 2018), rotavirus (Shahrudin et al., 2018), RSV (Chen et al., 2023), rabies virus (Franka et al., 2009), Zika virus (Sintara et al., 2025), and SARS-CoV-2 (Turan et al., 2021). Similarly, different eBeam modalities have shown viral inactivation and antigen preservation such as in case of 200 keV LEEB for influenza H3N8 (Fertey et al., 2016); 10 MeV HEEB for rotavirus (Skrobarczyk et al., 2022) and HIV-2/hepatitis A/pseudorabies/porcine parvovirus (Schmidt et al., 2012); and 4.5 MeV MEEB for murine norovirus-1 and Tulane virus (Predmore et al., 2015). However, these studies differ substantially in experimental conditions including sample matrix, irradiation temperature, dosimetry systems, and pathogen type making it difficult to assess a cross-modality comparison of viral inactivation efficiency.

To date, no published study has directly compared eBeam modalities (HEEB and MEEB) with gamma irradiation for viral inactivation under identical experimental parameters. Such empirical data is essential if eBeam is to be widely adopted in any format. To address this critical gap, the present study systematically compared the viral inactivation kinetics of HEEB (10 MeV), MEEB (4.5 MeV), and ^60^Co gamma irradiation using three human viral pathogens representing diverse structural and genomic composition. Human rotavirus (RV, a non-enveloped double stranded RNA virus of family Reoviridae), human respiratory syncytial virus type A2 (RSV, an enveloped single stranded negative sense RNA virus of family Pneumoviridae) and influenza A/PR/8/34/H1N1 virus (IAV, an enveloped, segmented single stranded negative sense RNA virus of family Orthomyxoviridae) were used for inactivation studies. Critical parameters such as sample matrix (PBS), sample packaging shipping temperature (4 °C), and dosimetry (alanine/EPR) were maintained across all modalities. LEEB was excluded as alanine dosimetry is incompatible with low-energy electrons and appropriate biocontainment during LEEB exposure was not feasible. The university’s biosafety guidelines did not allow for the direct exposure of the viral pathognes to LEEB in the laboratory setting without appropriate containment. Beyond inactivation kinetics, the functional characteristics of eBeam inactivated viruses were assessed. The impact of HEEB and MEEB irradiation on antigenic protein preservation were compared to traditional heat and formalin-inactivation methods across all three viruses (Lelie et al., 1987; Fan et al., 2015; Hankaniemi et al., 2019). Antigenic preservation after gamma irradiation was not assessed, as this has already been well documented for various viruses Franka et al., 2009; Salehi et al., 2018; Shahrudin et al., 2018; Turan et al., 2021; Chen et al., 2023; Sintara et al., 2025). We hypothesized that, when delivered at equivalent absorbed doses, high-energy (10 MeV) and medium-energy (4.5 MeV) electron beam irradiation would achieve viral inactivation efficiencies comparable to cobalt-60 gamma irradiation, while preserving viral antigenicity and virus–host receptor recognition more effectively than conventional heat- and formalin-based inactivation methods.

## 2 Materials and methods

### 2.1 Virus propagation

Human rotavirus (RV), human respiratory syncytial virus type A2 (RSV) and influenza A/PR/8/34/H1N1 virus (IAV) were used in this study. RV, RSV and IAV were propagated in MA104 (African green monkey fetal kidney epithelial cells; ATCC CRL-2378.1), HEp-2 (human laryngeal epidermoid carcinoma epithelial cells; ATCC CCL-23), and Madin-Darby Canine Kidney (MDCK; canine renal epithelial cells; ATCC CCL-34) cell lines, respectively. The cells lines were maintained in high-glucose Dulbecco’s Modified Eagle Medium (DMEM; Gibco, Thermo Fisher Scientific, NY) supplemented with 10% heat inactivated fetal bovine serum and 1% Penicillin–Streptomycin (Gibco, Thermo Fisher Scientific, NY). Virus activation and propagation were facilitated by TPCK-treated trypsin for RV and IAV at a final concentration of 1 μg/mL with all cultures maintained at 37 °C and 5% CO₂ incubator. To obtain high-titer viral stocks, culture supernatants were harvested and concentrated using 50 kDa molecular weight cut-off (MWCO) filters (MilliporeSigma, Saint Louis, MO). The concentrated viruses were diluted in PBS to defined concentrations and titers were estimated by 50% Tissue Culture Infectious Dose (TCID₅₀) assay using Reed and Muench (1938) method. Concentrated viral stocks with defined titers were used for all the analyses in the experimental workflow including (1) inactivation kinetics and D_10_ value determination, (2) complete virus inactivation, (3) antigen preservation, and (4) virus–host interactions as illustrated in Figure 1.

**Figure 1:**
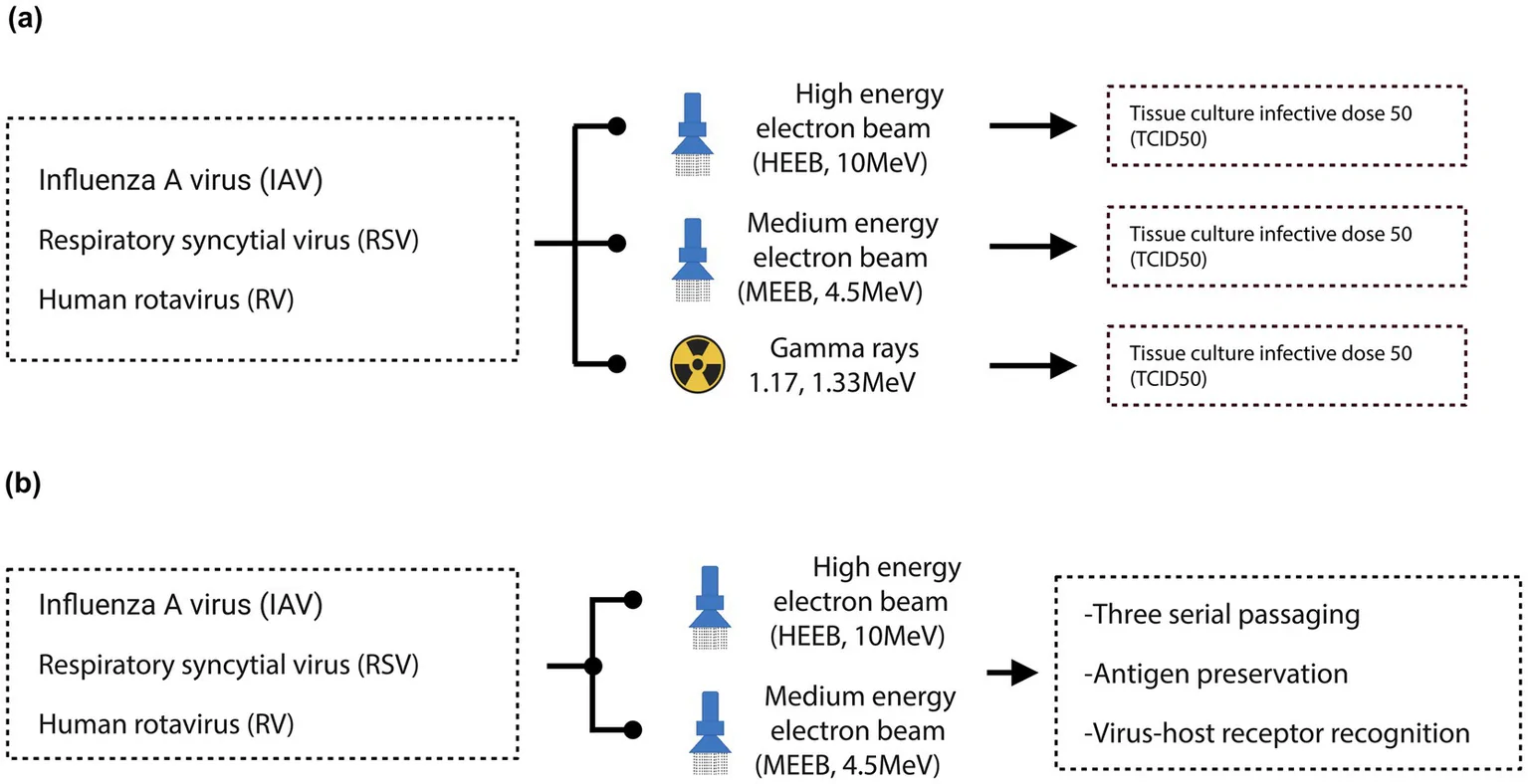
Schematic workflow of inactivation kinetics, complete viral inactivation, antigenic preservation, and virus-host interaction of the three human viruses. **(a)** Three human viruses were irradiated with defined doses (0, 2, 4, 6, 8, and 10 kGy) using electron beam (high-energy, and medium-energy) and cobalt-60 gamma radiation to determine D10 values. **(b)** At lethal doses (12, 15, 18, and 20 kGy), antigenic preservation, complete virus inactivation, and virus-host interactions were assessed across all three viruses.

### 2.2 Virus inactivation using HEEB, MEEB, and gamma irradiation

To evaluate the influence of radiation energy and dose rate on viral inactivation efficiency, three distinct ionizing radiation modalities were employed under standard biological conditions: High energy electron beam (HEEB), Medium energy electron beam (MEEB) and gamma radiation.

Defined titers of RV, RSV and IAV were packaged using a triple-layer containment system in compliance with TAMU IBC requirement for transportation of biohazardous materials. Samples were double-bagged using Whirl-Pak^®^ bags (Nasco, Fort Atkinson, WI) and subsequently placed in 95 kPa certified transport bags (Therapak^®^, Arlington Heights, IL). The packaged samples were maintained in blue ice (4 °C) during transportation to all irradiation facilities. Irradiation trials were scheduled to minimize the time between sample receipt, irradiation, and return to the TAMU laboratory for virus enumeration. HEEB treatment was performed at National Center for Electron Beam Research (NCEBR), Texas A&M University, College Station, TX using 10 MeV 15 kW S-band LINAC (L3 Pulse Sciences, Melbourne, FL). MEEB irradiation was conducted at 4.5 MeV utilizing the commercial irradiation services of EBeam Services, Inc. (Cranbury, NJ) and gamma irradiation was performed using ^60^Co source (1.77 and 1.33 MeV) at Steris AST (Libertyville, IL). The operational and physical parameters distinguishing the three irradiation modalities are summarized in Table 1. Defined titers of RV, RSV, and IAV were irradiated at incremental target doses of 0, 2, 4, 6, 8 and 10 kGy across all three radiation modalities. The inactivation kinetics experiments were performed in two completely independent trials with triplicate samples for each target dose point for HEEB, MEEB and gamma radiation.

**Table 1:** Physical and operational characteristics of the three ionizing radiation modalities- high-energy eBeam (HEEB), medium-energy eBeam (MEEB) and cobalt-60 based gamma irradiation, used for comparative viral inactivation.

Parameters	HEEB	MEEB	Gamma
Radiation Source	Linear Accelerator (S-band LINAC)	Linear accelerator (Dynamitron)	Cobalt-60 radioactive isotope
Radiation type	Accelerated electrons	Accelerated electrons	Photons (gamma rays)
Energy	10 MeV	4.5 MeV	1.17, 1.33 Mev
Power	15 kW	150 kW	—
Beam Current	1.5 mA	4.9–5.9 mA	—
Dose rate	3.84–4.31 kGy/s	0.37–0.45 kGy/s	0.00047–0.00142 kGy/s
Approximate processing time	Seconds	Seconds	hours
Dosimetry system	Alanine/EPR	Alanine/EPR	Alanine/EPR

Absorbed doses were measured with calibrated alanine dosimeters (GEX, Palm City, FL) using Bruker E-Scan ESR spectrometer (Bruker, Billerica, MA). The dose uniformity ratio (DUR = D_max_/D_min_) was determined for each irradiation configuration to confirm uniform dose delivery throughout the sample volume. DUR is a key parameter in radiation processing, as it indicates the uniformity of dose distribution throughout the entire sample and it is critically important in the determination of D_10_ values. A DUR value close to 1.0 signifies that minimum and maximum absorbed doses across the package were nearly equivalent. Alanine/EPR dosimetry was selected for this comparative study due to its established dose-rate independence, energy independence across the MeV range, minimal sensitivity to environmental variables, and long-term signal stability—properties essential for accurate cross-modality dose comparison (ISO/ASTM 51607, 2013). Post irradiation, samples were aliquoted and tested with minimal delay between irradiation and biological analysis to study the inactivation efficiency.

### 2.3 Inactivation kinetics and D_10_ value determination

Inactivation kinetics were determined for RV, RSV, and IAV across all three radiation modalities (HEEB, MEEB, and gamma) to enable direct cross-modality comparison of viral radiosensitivity. Surviving infectious virus populations at each target dose point were quantified using the TCID₅₀ assay as described earlier and titers expressed as log_10_ TCID_50_/mL. Viral survival curves were constructed by plotting log_10_ TCID_50_/mL as a function of absorbed dose (kGy). The D_10_ values were calculated as the negative reciprocal of the slope and reported as mean ± standard error (SE) derived from two independent experimental trials, each comprising three replicates per dose point.

### 2.4 Complete inactivation confirmation of eBeam inactivated viruses

To evaluate the complete viral inactivation, defined titers of viral suspensions were irradiated under both HEEB and MEEB modalities at lethal doses (target dose as 12, 15, 18, and 20 kGy). These doses were selected to exceed theoretical complete inactivation threshold calculated from D_10_ values. Complete inactivation was verified through three consecutive serial passages on susceptible cell monolayers (WHO, 2011). About 2 mL of irradiated and control samples (representing ~40% of total irradiated volume) was inoculated onto 80–90% confluent cell monolayers of corresponding cell line to monitor cytopathic effects (CPEs) using T75 cell culture flask. The loss of viral infectivity was tested in triplicate for each dose point. Regardless of CPE observation, the infected cells underwent a freeze–thaw cycle, after which entire culture supernatant was harvested, centrifuged and concentrated using 50 kDa MWCO (Sigma-Aldrich, Saint Louis, MO) filters to 200 μL and the entire volume passaged onto fresh cell monolayers. This serial passage process was repeated through three consecutive passages (P1, P2, and P3) for all HEEB and MEEB irradiated samples with three replicates at each dose point. Complete inactivation was defined as the absence of detectable CPE across all three serial passages in all three replicates at a given dose.

In parallel, chemical and thermal inactivation using formalin and heat, respectively, were performed to generate conventionally inactivated viral preparations as comparative controls for subsequent analyses. For chemical inactivation, formalin (Sigma-Aldrich, Saint Louis, MO; Cat. No. F1635-25) was used to inactivate all three tested viruses following established protocols. RV was treated with 2% (v/v) formalin and incubated at 25 °C for 30 min, followed by 37 °C for 30 min (Skrobarczyk et al., 2022). RSV was inactivated using 0.025% (v/v) formalin at 37 °C for 96 h (Bayer et al., 2018) and IAV culture supernatants were incubated with 0.1% (v/v) formalin at 4 °C for 16 h (Fertey et al., 2016). For thermal inactivation, all three viruses were exposed to 85 °C for 20 min (Bayer et al., 2018).

### 2.5 Antigenic preservation assessment of eBeam inactivated viruses

To assess whether eBeam inactivation preserves immunodominant surface proteins, the antigenic integrity of eBeam (HEEB and MEEB) inactivated viral preparations was compared against untreated (non-irradiated), heat inactivated and formalin inactivated controls. Key surface antigens- VP4 (RV), Fusion (F) protein (RSV), and hemagglutinin/HA (IAV) were quantified using indirect ELISA, direct ELISA, and hemagglutination assay (Bonneux et al., 2024; Kelleher et al., 2025).

#### 2.5.1 VP4 antigenicity of rotavirus (indirect ELISA)

The integrity of VP4 outer capsid protein on eBeam inactivated RV was assessed by indirect ELISA. Fifty microliters (50 μL) of inactivated virus preparations were mixed 1:1 (v/v) with carbonate–bicarbonate coating buffer (0.05 M, pH 9.6) and coated onto 96-well polystyrene ELISA plate. The plate was incubated overnight at 37 °C and subsequently washed with PBS (phosphate buffered saline) thrice to remove the unbound antigen. Unspecific binding sites in the wells were blocked with non-fat dry milk for 1 h at room temperature, followed by three washing with PBST (phosphate buffered saline with 0.05% Tween 20). A 100 μL solution of 1:1000 diluted rabbit polyclonal anti-VP4 antibody (MyBioSource, San Diego, CA, United States; Cat. No. MBS7151952) was added to each well and incubated for 1 h, followed by three washing with PBST to remove unbound antibody. The antibody dilution was selected based on prior optimization experiments using different dilutions (1:500, 1:1,000, 1:200) were tested to achieve best signal to noise ratio. HRP-conjugated goat anti-rabbit IgG secondary antibody (Invitrogen™, Thermo Fisher Scientific, Waltham, MA, USA; Cat. No. A16110) was added at a dilution of 1:1,000 and incubated for 1 h. After final three washes, TMB substrate was applied and incubated for 30 min, and the optical density was measured at 450 nm to determine VP4 antigenicity.

#### 2.5.2 Fusion (F) protein antigenicity of RSV (direct ELISA)

The structural integrity of the RSV fusion (F) glycoprotein following eBeam inactivation was assessed by direct ELISA. Fifty microliters (50 μL) of HEEB and MEEB inactivated viral preparations were mixed with coating buffer (1:1 ratio) and coated on 96-well microplates followed by incubation at 37 °C overnight. To avoid non-specific binding, the wells were blocked with non-fat dry milk for 1 h at ambient temperature, followed by additional three washes using PBST. Subsequently, an HRP-conjugated anti-RSV-F rabbit antibody (Sino Biological, Beijing, China; Cat. No. 11049-R302-H) was added using optimized 1:2000 dilution and incubated for 60 min. Unbound antibodies were removed with three PBST washes, and then TMB substrate solution was added followed by stop solution. The optical density was measured at 450 nm.

#### 2.5.3 Hemagglutinin (HA) antigenicity of IAV (hemagglutinin assay)

Preservation of the influenza HA protein following eBeam inactivation was evaluated by the hemagglutination (HA) assay, which measures the functional capacity of HA to agglutinate erythrocytes. HEEB and MEEB-inactivated virus preparations (50 μL) were subjected to two-fold serial dilutions across 96 well V-bottom microtiter plates. Untreated (non-irradiated) virus served as a positive control, and PBS was used as the negative control. Fifty microliters of turkey erythrocyte suspension (0.5%) (v/v) were added to each well, and plates were incubated for 30 min at room temperature until the erythrocytes settled in the positive control well (Ali et al., 2018). The assay measures the ability of HA protein agglutination with the turkey’s red blood cells. Wells were assessed visually for the presence or absence of hemagglutination, and the HA titer was recorded as the reciprocal of the highest dilution exhibiting complete hemagglutination (WHO, 2011).

### 2.6 Virus-host interaction

To evaluate whether eBeam-inactivated viruses retain functional virus-host recognition, HEEB- and MEEB-inactivated preparations at lethal doses (12, 15, 18, and 20 kGy) were assessed for their ability to adsorb onto susceptible host cell monolayers and subsequently detected using virus-specific antibodies through cell-based ELISA. For this, 50 μL of the IAV and RSV were allowed to adsorb overnight on the 80–90% confluent MDCK and HEp-2 cells, respectively. Following incubation, non-adsorbed viruses were removed by gentle washing, and cells were fixed with chilled acetone (80%) to preserve cell morphology and virus-receptor complexes. Fixed monolayers were blocked with 5% non-fat dry milk for 1 h at room temperature to prevent non-specific antibody binding, followed by three PBST washes. For HA detection, HRP conjugated anti-HA antibody, mouse monoclonal (Sino Biological Inc., Beijing, China; Cat. No. 11684-MM03-H) was added in 1:2,000 dilution to MDCK cells. For F- protein detection, HRP-conjugated anti-RSV-F rabbit antibody (Sino Biological, Beijing, China; Cat. No. 11049-R302-H) was added at 1:2,000 dilution to Hep-2 cells and incubated for 60 min. Subsequently, TMB substrate solution was added, and the optical density was measured to evaluate the binding of the irradiated virus to host receptors. For human rotavirus, HEEB and MEEB irradiated viruses were tested through indirect ELISA. In this procedure, 50 μL of the irradiated virus was plated onto MA104 cells and incubated overnight to allow viral attachment in the presence of TPCK trypsin (1μg/mL). Afterward, the cells underwent antigen fixation, and blocking was performed as mentioned earlier. A primary anti-VP4 antibody (1:1,000 dilution) was then applied and incubated for 60 min, followed by three PBST washes. The HRP-labeled secondary antibody (1:1,000 dilution) was added and incubated for an additional hour, after which the wells were washed three more times. Finally, TMB substrate was added, and the optical density was measured to determine the extent of viral attachment to host cell receptors.

### 2.7 Data analysis

The D_10_ values were determined from linear regression of virus surviving curves plotted as a function of absorbed doses and calculated as the negative reciprocal of the regression slope. Goodness of fit was assessed by *R*^2^ and D_10_ were reported as mean ± standard error from two independent trials each comprising of three replicates per dose point. For statistical comparisons across different radiation modalities, one-way analysis of variance (ANOVA) followed by Tukey’s multiple comparisons test was employed. Significance for D_10_ comparisons across radiation modalities was defined at *p* < 0.01 to provide conservative interpretation of inter-modality differences. Linear regression and statistical analyses were performed using GraphPad Prism (version 11.0.1; GraphPad Software Inc., San Diego, CA, United States).

## 3 Results

### 3.1 Virus inactivation kinetics and D_10_ value determination

To determine the effect of irradiation modalities on viral inactivation efficiency, inactivation kinetics was studied across HEEB, MEEB and gamma for three structurally and genomically diverse human viruses: RV, RSV and IAV. Linear regression analysis of log_10_ surviving fraction of viruses versus absorbed doses demonstrated first order inactivation kinetics for all the virus-radiation modality combination with *R*^2^ > 0.95 (Figure 2, SI 1). The D_10_ values representing the radiation dose required for 90% virus inactivation across HEEB, MEEB and gamma radiation are summarized in Table 2.

**Figure 2:**
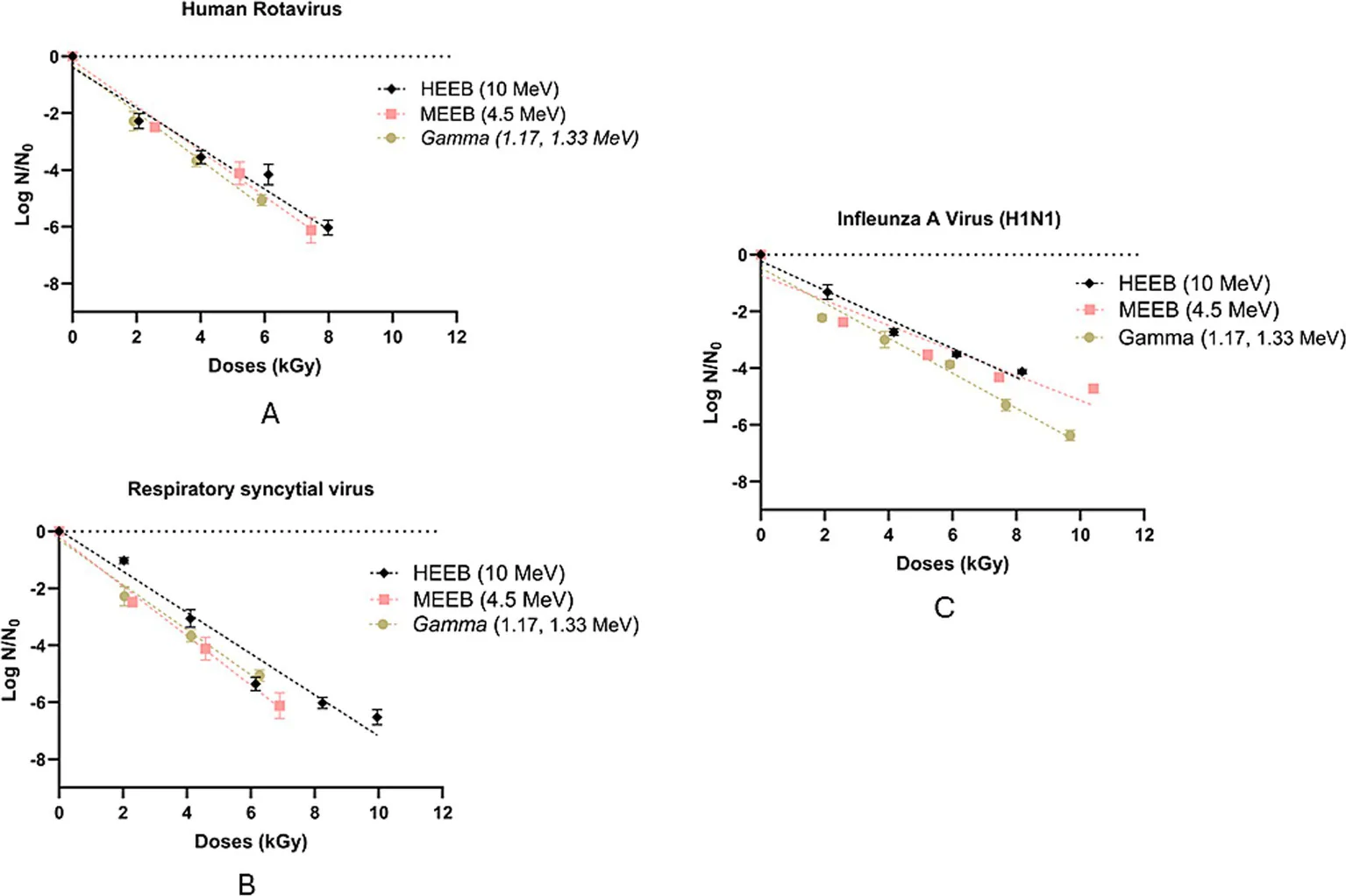
Cross-modality comparison of viral inactivation kinetics under high-energy eBeam, medium-energy eBeam, and gamma irradiation. Logarithmic reduction (Log N/N_0_) in survival populations of **(A)** human rotavirus (RV), **(B)** human respiratory syncytial virus (RSV), and **(C)** influenza A virus (IAV) following high-energy electron beam (HEEB- 10 MeV), medium-energy electron beam (MEEB- 4.5 MeV), and cobalt-60 based gamma (1.17, 1.33 MeV) irradiation.

**Table 2:** Comparative D_10_ values (kGy) for human rotavirus, respiratory syncytial virus, and influenza A virus following high-energy eBeam (10 MeV), medium-energy eBeam (4.5 MeV) irradiation and cobalt-60 based gamma irradiation (1.17, 1.33 MeV).

Virus	Decimal reduction dose (D₁₀) values (kGy)		
	HEEB (10 MeV)	MEEB (4.5 MeV)	Gamma (1.17, 1.33 MeV)
Human Rotavirus	1.25 ± 0.25^a^	1.29 ± 0.22^a^	1.23 ± 0.01^a^
Respiratory Syncytial Virus	1.45 ± 0.02^a^	1.73 ± 0.23^a^	1.03 ± 0.07^a^
Influenza A virus	1.95 ± 0.04^a^	2.27 ± 0.01^b^	1.62 ± 0.03^c^

D_10_ values are represented as Mean± Standard Error.

Different superscript letters (a, b, c) within a row indicate significant differences in D_10_ values across different irradiation modalities (ANOVA, *p* < 0.01).

Among the three viruses, RV was most radiosensitive with D_10_ value of 1.23 ± 0.01 (gamma), 1.25 ± 0.25 (HEEB), and 1.29 ± 0.22 kGy (MEEB), with no significant differences between modalities. Similarly, RSV exhibited D_10_ value of 1.03 ± 0.07 (gamma), 1.45 ± 0.02 (HEEB), and 1.73 ± 0.23 kGy (MEEB) with no significant difference (*p* > 0.01) between radiation modalities. In contrast, IAV was found to be relatively radioresistant, with D_10_ values of 1.62 ± 0.003 (gamma), 1.95 ± 0.04 (HEEB), and 2.27 ± 0.01 kGy (MEEB), with all pairwise comparisons significantly different for each of the radiation modality (Table 2). Results indicate that regardless of modality, the radiosensitivity hierarchy remained consistent (RV > RSV > IAV), indicating that virus-intrinsic properties also contribute in radiation sensitivity. Modality specific parameters including dose rate and energy exerted a secondary and virus-dependent influence, with inter modality difference emerging only for the relatively radioresistant IAV.

### 3.2 Complete inactivation confirmation of eBeam inactivated viruses

Three consecutive cell culture passages of eBeam (HEEB and MEEB), heat, and formalin inactivated virus samples were performed to confirm the complete loss of infectivity of three viruses at each target dose (12 kGy, 15 kGy, 18 kGy, and 20 kGy) in triplicates. Complete inactivation was defined as the absence of detectable CPEs across all three serial passages in all three replicates at a given dose. Results from the HEEB and MEEB inactivation studies are summarized in Table 3.

**Table 3:** Determination of the complete inactivation dose point following high-energy eBeam irradiation (10 MeV) and medium energy eBeam (4.5 Mev) through three serial passages for human rotavirus, human respiratory syncytial virus, and human influenza A virus.

Virus	Titer (Log TCID_50_/mL)	Treatment modality	Measured dose (kGy)	Serial passaging			Inactivation status
				P1	P2	P3	
Human rotavirus	10.18	Untreated		+	+	+	Incomplete
	10.18	HEEB	12.31	−	+	+	Incomplete
			15.56	−	−	+	Incomplete
			18.17	−	−	−	Complete
			20.73	−	−	−	Complete
	9.44	MEEB	13.29	−	+	+	Incomplete
			16.21	−	−	+	Incomplete
			19.81	−	−	−	Complete
			21.65	−	−	−	Complete
	10.18	Formalin		−	−	−	Complete
	10.18	Heat		−	−	−	Complete
Respiratory syncytial virus	6.70	Untreated		+	+	+	Incomplete
	6.70	HEEB	12.31	−	−	+	Incomplete
			15.56	−	−	−	Complete
			18.17	−	−	−	Complete
			20.73	−	−	−	Complete
	8.74	MEEB	13.29	−	−	−	Complete
			16.21	−	−	−	Complete
			19.81	−	−	−	Complete
			21.65	−	−	−	Complete
	6.70	Formalin		−	−	−	Complete
	6.70	Heat					Complete
Influenza A Virus (A/PR/8/H1N1)	9.44	Untreated		+	+	+	Incomplete
	9.44	HEEB	12.31	−	−	−	Complete
			15.56	−	−	−	Complete
			18.17	−	−	−	Complete
			20.73	−	−	−	Complete
	6.64	MEEB	13.29	−	−	−	Complete
			16.21	−	−	−	Complete
			19.81	−	−	−	Complete
			21.65	−	−	−	Complete
	9.44	Formalin		−	−	−	Complete
	9.44	Heat		−	−	−	Complete

Both HEEB and MEEB achieved complete inactivation of all three viruses, however, the minimum dose required for complete inactivation varied depending upon the virus titer rather than modality. For HEEB, complete inactivation was confirmed at 18.17 kGy for RV (initial titer: 10.18 log_10_ TCID_50_/mL), 15.56 kGy for RSV (initial titer: 6.7 log_10_ TCID_50_/mL), and 12.31 kGy for IAV (initial titer: 9.44 log_10_ TCID_50_/mL). For MEEB, complete inactivation was achieved at 19.81 kGy for RV (initial titer: 9.44 log_10_ TCID_50_/mL), and 13.29 kGy for both RSV (initial titer: 8.74 log_10_ TCID_50_/mL) and IAV (initial titer: 6.64 log_10_ TCID_50_/mL). Formalin and heat inactivation controls confirmed complete inactivation across all three passages for all viruses.

Notably, post-irradiation testing of human rotavirus (HEEB at 12.3 kGy and MEEB at 13.29 kGy) yielded negative results in the first passage, however, infectivity was detected in the second passage. Additionally, RV samples irradiated with HEEB at 15.56 kGy and MEEB at 16.21 kGy, as well as RSV samples exposed to HEEB at 12.3 kGy, tested negative across the initial two passages but infectivity was detected in the third passage (Table 3). These observations underscore the importance of conducting three consecutive passages to reliably confirm complete viral inactivation.

### 3.3 Antigenic preservation assessment of eBeam inactivated viruses

Across all three viruses, HEEB and MEEB inactivation consistently preserved the antigens comparable to untreated viruses, while substantial antigen loss was observed in case of heat and formalin inactivated viruses (Figure 3). In RV, VP4 surface protein showed no significant difference between untreated controls and eBeam inactivated viral suspension at lethal doses. In contrast, heat inactivation resulted in 5.09 to 6.56-fold reduction, and formalin inactivation caused 1.69 to 1.72-fold reduction in VP4 antigenicity compared to HEEB and MEEB (Figures 3a,d). Similarly, surface fusion-protein of RSV showed no significant difference between untreated virus to HEEB lethal doses (15.56, 18.17, 20.73 kGy) and MEEB lethal doses (13.29, 16.20, 19.81, 21.65 kGy). Conversely, a 5.79 to 7.89-fold reduction was noted for the heat-inactivated RSV virus, and a 1.26 to 1.72-fold reduction for the formalin-inactivated RSV relative to the HEEB and MEEB-inactivated samples (Figures 3b,e). Influenza A virus was assessed for the hemagglutinin antigen integrity by its ability to bind with turkey red blood cells. The results showed no difference in hemagglutinin antigen integrity between untreated virus to HEEB lethal doses (12.31, 15.56, 18.17, 20.73 kGy) and MEEB lethal doses (13.29, 16.20, 19.81, 21.65 kGy). In comparison, results showed significant differences with 256-fold reduction for heat-inactivated and 2-fold reduction for formalin-inactivated influenza A virus suspensions (Figures 3c,f).

**Figure 3:**
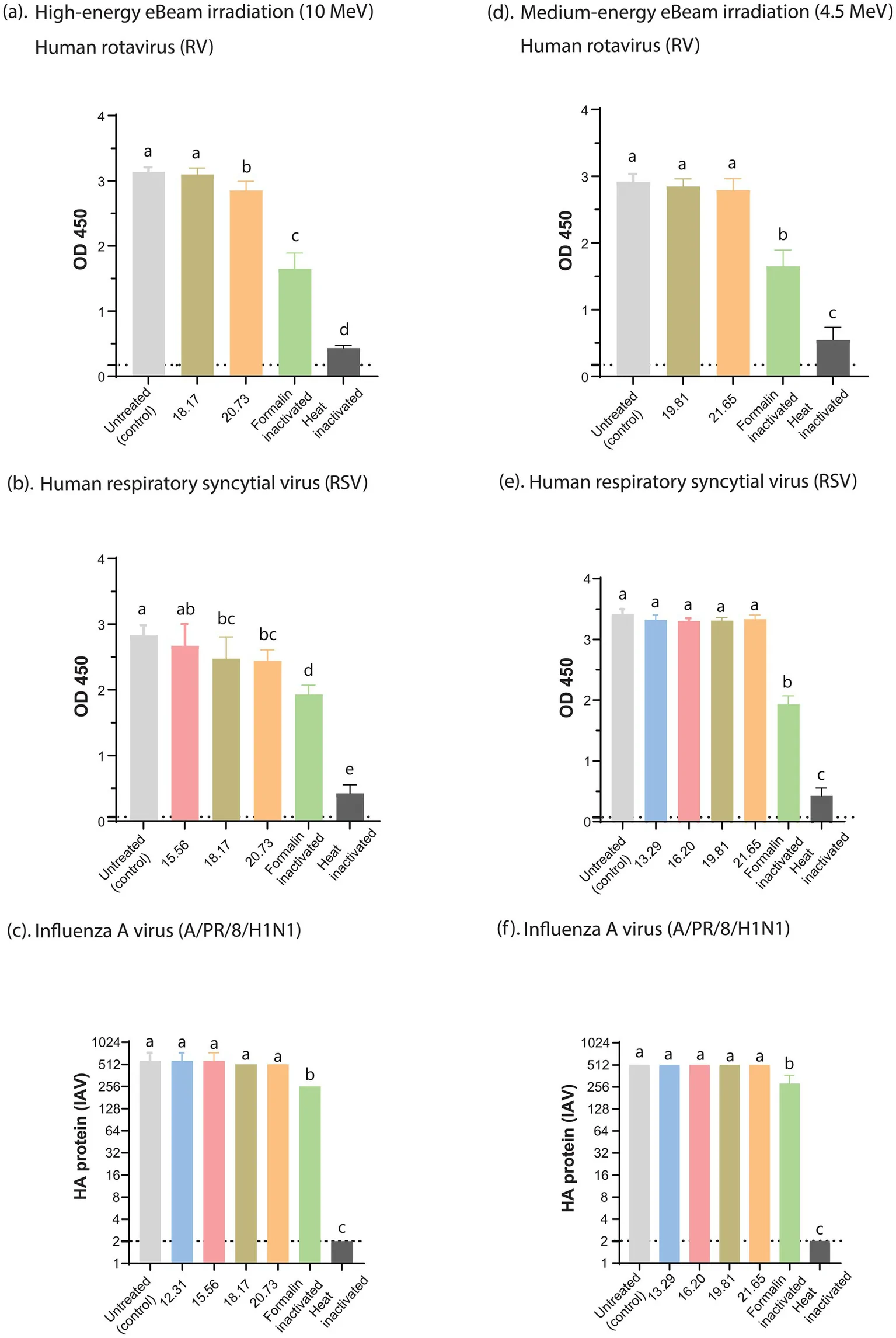
Antigenic preservation of immunodominant surface proteins following high-energy eBeam (HEEB; 10 MeV) and medium-energy eBeam (MEEB; 4.5 MeV) inactivation compared to heat and formalin-inactivated controls across three human viruses. VP4 outer capsid protein of human rotavirus (RV) following **(a)** HEEB and **(d)** MEEB irradiation at lethal doses assessed by indirect ELISA (OD₄₅₀). Fusion (F) glycoprotein of human respiratory syncytial virus (RSV) following **(b)** HEEB and **(e)** MEEB irradiation assessed by direct ELISA (OD₄₅₀). Hemagglutinin (HA) protein of influenza A virus (A/PR/8/H1N1) following **(c)** HEEB and **(f)** MEEB irradiation assessed by hemagglutination assay (HA titer, log₂ scale). Different alphabetical letters above the histogram denote significant differences between groups (one-way ANOVA, Tukey’s multiple comparisons test, *p* < 0.01).

These results demonstrate that eBeam treatment (HEEB and MEEB) preserve the native confirmation and structural integrity of the key antigenic proteins even at doses substantially exceeding the complete inactivation threshold, while heat and formalin inactivation compromises the antigenic integrity across all viral surface proteins tested.

### 3.4 Virus-host interaction

The HEEB and MEEB irradiated viral suspensions which showed complete inactivation and preserved antigenic proteins were further evaluated for their ability to be recognized by their respective host’s receptors. Figure 4 shows that both HEEB and MEEB inactivation demonstrated enhanced virus-host interaction than heat or formalin-inactivated samples across all three tested viruses. The eBeam-inactivated RV displayed significant difference (*p* < 0.0001) with 1.85-fold (HEEB) and 1.82- fold (MEEB) better virus-host recognition than heat-inactivated samples and 0.89-fold (HEEB) and 1.14-fold (MEEB) better host recognition than formalin-inactivated samples (Figure 4A). However, the untreated control RV preparation exhibited 1.07- to 1.18-fold higher virus-host activity of the VP4 protein compared to HEEB and MEEB inactivated samples. Similar results were observed for RSV, where eBeam inactivation resulted in 1.58 to 1.54-fold and 1.67 to 1.63-fold greater virus-host interaction of fusion-protein (*p* < 0.0001) in comparison to heat and formalin-inactivated samples, respectively, (Figure 4B). For IAV, lethal eBeam treatment demonstrated 23.03 to 23.11-fold and 1.29 to 1.31-fold better virus-host interaction of hemagglutinin protein with statistical significance (*p* < 0.0001) in comparison to heat and formalin-inactivated influenza A virus, respectively, (Figure 4C). These findings emphasize the superior preservation of functional virus-host interactions following eBeam (HEEB and MEEB) inactivation.

**Figure 4:**
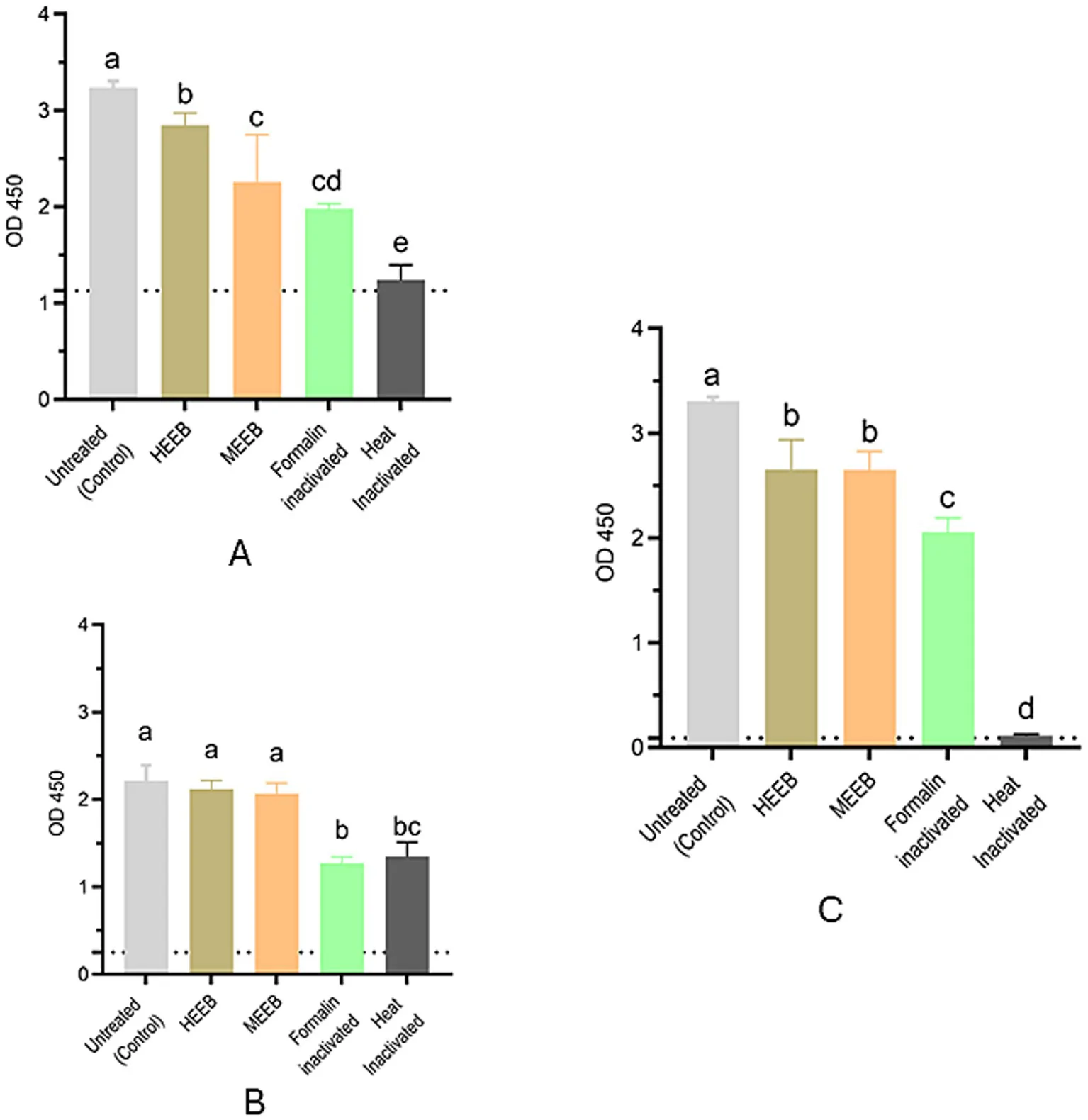
Virus-host receptor interaction of eBeam-inactivated (HEEB and MEEB), formalin-inactivated, heat-inactivated, and untreated (control) viral preparations across three human viruses. **(A)** VP4-mediated binding of human rotavirus to MA104 host cells assessed by indirect cell-based ELISA (OD₄₅₀). **(B)** Fusion (F) protein-mediated binding of human respiratory syncytial virus to HEp-2 host cells assessed by direct cell-based ELISA (OD₄₅₀). **(C)** Hemagglutinin (HA)-mediated binding of influenza A virus (A/PR/8/H1N1) to MDCK host cells assessed by cell-based ELISA (OD₄₅₀). Different alphabetical letters above the histogram denote significant differences between groups (one-way ANOVA, Tukey’s multiple comparisons test, *p* < 0.01).

## 4 Discussion

This study provides the first systematic cross modality comparison between accelerator-based (eBeam: HEEB and MEEB) and radioactive isotope-based (gamma) radiation for viral inactivation. Previous studies investigated viral inactivation using individual radiation modalities: rotavirus with HEEB (Skrobarczyk et al., 2022), RSV with LEEB (Bayer et al., 2018), and influenza A with LEEB and gamma (Fertey et al., 2016), but employed different matrices, temperatures, or dosimetry systems, precluding reliable cross-modality comparison of D_10_ values. Present study addresses this gap by maintaining standard parameters such as sample matrix (PBS), unified alanine/EPR dosimetry system (ISO/ASTM 51607, 2013) and maintaining the temperature (4 °C) during sample handling and transportation, while varying the radiation modality enabling direct comparison of inactivation efficiency between HEEB, MEEB and gamma irradiation.

The primary physical differences between these modalities as highlighted in Table 1 are dose rate (HEEB: 3.84–4.31 kGy/s; MEEB: 0.37–0.45 kGy/s; Cobalt-60: 1.7–5.1 kGy/h) and particle energy (HEEB: 10 MeV electrons; MEEB: 4.5 MeV electrons; gamma: 1.17 and 1.33 MeV uncharged photons). We acknowledge that beam current and dose rate could not be experimentally normalized across eBeam modalities, as irradiations were necessarily performed at physically distinct facilities. Nevertheless, the consistent patterns observed across all three viruses suggest systematic modality-dependent effects rather than experimental variation.

Results demonstrate that all three radiation modalities effectively inactivated the tested viruses with D_10_ values ranging from 1.03 ± 0.07 to 2.27 ± 0.01 kGy. The key finding from this study is that the eBeam modalities (HEEB and MEEB) achieve viral inactivation efficiency comparable to gamma radiation. Two of the three tested viruses (RV and RSV) demonstrated no statistically significant (*p* > 0.01) difference in D_10_ values across HEEB, MEEB and gamma despite the substantial difference in dose rate and energy between sources (Table 2). This finding is consistent with previous studies on bacterial inactivation by Tallentire et al. (2010), who compared *Bacillus pumilus* spore inactivation using ^60^Co gamma and 10 MeV eBeam and reported statistically indistinguishable D_10_ values, and Tallentire and Miller (2015), extended this comparison to include 200 keV LEEB and again found no significant modality-dependent differences. McEvoy et al. (2023) conducted a comprehensive study and showed equal bactericidal effectiveness across X-ray, gamma and eBeam in industrial sterilization of medical devices. The present study extends this principle of modality equivalence specifically to viral inactivation in aqueous biological matrices.

However, statistically significant D_10_ values were observed for IAV across all three modalities (*p* < 0.01), which could be related to the virus’ intrinsic radioresistance to inactivation rather than modality specific effect. Within the three tested viruses, a consistent radiation sensitivity hierarchy was observed with RV > RSV > IAV across all the modalities. Ionizing radiation induced viral inactivation is governed by both direct ionization of viral nucleic acids and indirect effects mediated by reactive species generated through water radiolysis. Variations in electron energy, and consequently linear energy transfer (LET), influence the spatial distribution and yield of these reactive species, thereby impacting viral genome damage and preservation of antigenic structures (Buxton et al., 1988; Jonah et al., 1990; Fertey et al., 2016). Based on the classical target theory (Lea, 1946; Pollard, 1953), inactivation probability is directly proportional to the radiation sensitive target volume within the irradiated entity. Consequently, D_10_ is inversely proportional to genome size, as larger genome presents proportionally greater chances of radiation induced ionization events per unit absorbed dose (Lea, 1946; Praveen et al., 2013; Feldmann et al., 2019). Thus, higher susceptibility of RV could be attributable to the relatively large genome size (18.5 kb ds RNA) compared to RSV (15.2 kb ssRNA) and IAV (13.5 kb segmented ssRNA).

The D_10_ value (1.95 ± 0.04 kGy) for the IAV (A/PR/8/34/H1N1) using PBS matrix in current study is comparable to previously reported 2.3 kGy for IAV (A/Chicken/TX/02/rH5N3) using HEEB irradiation (Brahmakshatriya et al., 2009). However, rotavirus D_10_ value (1.25 ± 0.25 kGy) was found lower than the previously reported D_10_ value (2.38 kGy) with HEEB irradiation (Skrobarczyk et al., 2022). This suggests that the D_10_ value is influenced by factors beyond ionizing radiation energy or genome size, including the virus strain, matrix composition and other variables. However, the decrease in log_10_ titers across all three tested viruses showed an inverse but linear relationship, demonstrating the efficiency of high and medium energy eBeam technology (Figure 2). Both HEEB and MEEB demonstrated effective viral inactivation, establishing their suitability as alternatives to ^60^Co gamma radiation. Electron beam modalities offer significant operational advantage of achieving complete viral inactivation and achieve sterility assurance level (SAL) in a fraction of the time without producing radioactive emissions (Gaidamakova et al., 2012).

Both HEEB and MEEB irradiation showed significantly better preservation of surface antigenic proteins and virus-host interaction compared to traditional chemical-inactivation method, which is widely used in vaccine development but often leads to reduced antigenic response due to damaged antigenic proteins (Fertey et al., 2020). Chemical inactivation is the method used for the FDA-approved influenza inactivated vaccine since 2013 in the United States, which is time-consuming and complicates downstream assays. Across all three viruses, eBeam-inactivated preparations-maintained antigenicity equivalent to untreated virus even at doses up to 21.65 kGy—substantially exceeding complete inactivation thresholds—while heat inactivation caused 5.09–256-fold loss and formalin produced 1.26–2-fold reduction. These results are consistent with previous reports (Fertey et al., 2016; Skrobarczyk et al., 2022) and reflect the fundamental mechanistic advantage of radiation-based inactivation: preferential genomic destruction through nucleic acid targeting while leaving structural proteins largely intact at the doses required for inactivation.

Antigenic preservation was further validated by virus-host interaction assays, where eBeam inactivated viruses displayed superior recognition to host-receptors compared to heat and chemical-inactivated viruses. Unlike the non-replicative eBeam-treated samples, untreated control viruses demonstrated stronger interactions, likely reflecting ongoing viral replication. Compared to chemical inactivation that produce narrower antibody-specific responses (Singleton et al., 2021; Wijewardana et al., 2023), irradiated vaccines using eBeam and gamma modalities can offer broader and cellular immune responses (Alsharifi et al., 2009; Furuya et al., 2010; David et al., 2019; Wijewardana et al., 2023; Schulze et al., 2024; Belay et al., 2025). Overall, these results highlight eBeam irradiation as a promising alternative for future vaccine development and biopharmaceutical applications due to its rapid inactivation and superior antigenic preservation of vaccinal target sites.

The differential effects of ionizing radiation (eBeam and gamma), heat, and formalin on viral infectivity and antigen preservation can be attributed to the inherent differences in how these three viral inactivation methodologies function. Ionizing radiation functions by breakage of the phosphor-diester bonds on the nucleic acid molecule while formalin functions by creating covalent crosslinks in and between proteins and nucleic acids whereas heat treatment induces protein unfolding, aggregation and other irreversible thermal damage (Ward, 1988; Hoffman et al., 2015; Loveday et al., 2021).

## 5 Conclusion

This study provides the first systematic comparison of HEEB (10 MeV), MEEB (4,5 MeV) and Cobalt-60 gamma irradiation for viral inactivation under standardized conditions of sample matrix, shipping temperature and alanine dosimetry system. All three modalities effectively inactivated human rotavirus, respiratory syncytial virus and influenza A virus. The inactivation kinetics studies demonstrated that viral inactivation efficiency achieved by eBeam modalities are comparable to gamma irradiation. Therefore, HEEB and MEEB represents sustainable nonradioactive alternatives to gamma radiation, capable of achieving effective viral inactivation in seconds compared to hours required for gamma, owing to its high dose rate. Critically, eBeam inactivation preserved key immunodominant surface proteins (VP4, F-protein and HA) at levels equivalent to untreated control even at lethal dose of up to 20.73 kGy (HEEB) and 21.65 kGy (MEEB), substantially outperforming heat and formalin-inactivated virus preparations. Also, eBeam inactivated viruses maintained superior functional virus-host receptor recognition indicating the functional integrity of antigenic proteins. Collectively, these findings establish eBeam technology as a promising platform for effective viral inactivation for biopharmaceutical applications, combining rapid and complete inactivation with superior antigenic preservation and functional host receptor recognition essential for immunological efficacy.

## Data Availability

The original contributions presented in the study are included in the article/Supplementary material, further inquiries can be directed to the corresponding author.

## References

[ref1] Ali M. Yaqub T. Mukhtar N. Imran M. Ghafoor A. Shahid M. F. . (2018). Prevalence and phylogenetics of H9N2 in backyard and commercial poultry in Pakistan. Avian Dis. 62:416. doi: 10.1637/11690-062117-ResNote.1, 31119926

[ref2] Alsharifi M. Furuya Y. Bowden T. R. Lobigs M. Koskinen A. Regner M. . (2009). Intranasal flu vaccine protective against seasonal and H5N1 avian influenza infections. PLoS One 4:e5336. doi: 10.1371/journal.pone.0005336, 19401775 PMC2671162

[ref3] Bayer L. Fertey J. Ulbert S. Grunwald T. (2018). Immunization with an adjuvanted low-energy electron irradiation inactivated respiratory syncytial virus vaccine shows immunoprotective activity in mice. Vaccine 36, 1561–1569. doi: 10.1016/j.vaccine.2018.02.014, 29439869

[ref4] Belay E. Bitew M. Ibrahim S. M. Dessalegn B. Abey S. L. Dejene H. . (2025). Gamma-irradiated fowl cholera vaccines formulated with different adjuvants induced antibody response and cytokine expression in chickens. Front. Immunol. 16:1513443. doi: 10.3389/fimmu.2025.1513443, 40103817 PMC11914910

[ref5] Bhatia S. S. Pillai S. D. (2022). Ionizing radiation Technologies for Vaccine Development - a Mini review. Front. Immunol. 13:845514. doi: 10.3389/fimmu.2022.845514, 35222438 PMC8873931

[ref6] Bonnafous P. Nicolaï M. C. Taveau J. C. Chevalier M. Barrière F. Medina J. . (2014). Treatment of influenza virus with Beta-propiolactone alters viral membrane fusion. Biochim. Biophys. Acta Biomembr. 1838, 355–363. doi: 10.1016/j.bbamem.2013.09.021, 24140008

[ref7] Bonneux B. Jacoby E. Ceconi M. Stobbelaar K. Delputte P. Herschke F. (2024). Direct-acting antivirals for RSV treatment, a review. Antivir. Res. 229:105948. doi: 10.1016/j.antiviral.2024.105948, 38972604

[ref8] Brahmakshatriya V. Lupiani B. Brinlee J. L. Cepeda M. Pillai S. D. Reddy S. M. (2009). Preliminary study for evaluation of avian influenza virus inactivation in contaminated poultry products using electron beam irradiation. Avian Pathol. 38, 245–250. doi: 10.1080/03079450902912150, 19468943

[ref9] Butreddy A. Janga K. Y. Ajjarapu S. Sarabu S. Dudhipala N. (2021). Instability of therapeutic proteins — an overview of stresses, stabilization mechanisms and analytical techniques involved in lyophilized proteins. Int. J. Biol. Macromol. 167, 309–325. doi: 10.1016/j.ijbiomac.2020.11.188, 33275971

[ref10] Buxton G. V. Greenstock C. L. Helman W. P. Ross A. B. (1988). Critical review of rate constants for reactions of hydrated electrons, hydrogen atoms and hydroxyl radicals (OH/O− in aqueous solution). J. Phys. Chem. Ref. Data 17, 513–886. doi: 10.1063/1.555805

[ref11] Chen F. Park H.-R. Ji H. J. Kwon Y. Kim M.-K. Song J. Y. . (2023). Gamma irradiation-inactivated respiratory syncytial virus vaccine provides protection but exacerbates pulmonary inflammation by switching from Prefusion to Postfusion F protein. Microbiol. Spectrum 11:e01358–23. doi: 10.1128/spectrum.01358-23, 37272801 PMC10434263

[ref12] Cleland M. R. (2006) Industrial Applications of electron Accelerators CAS 2005 - CERN Accelerator School: Small Accelerators, Proceedings. Zeegse, Netherlands: CERN Accelerator School, 383–416.

[ref13] David S. C. Norton T. Tyllis T. Wilson J. J. Singleton E. V. Laan Z. . (2019). Direct interaction of whole-inactivated influenza a and pneumococcal vaccines enhances influenza-specific immunity. Nat. Microbiol. 4, 1316–1327. doi: 10.1038/s41564-019-0443-4, 31110357

[ref14] Delgado M. F. Coviello S. Monsalvo A. C. Melendi G. A. Hernandez J. Z. Batalle J. P. . (2009). Lack of antibody affinity maturation due to poor toll-like receptor stimulation leads to enhanced respiratory syncytial virus disease. Nat. Med. 15, 34–41. doi: 10.1038/nm.1894, 19079256 PMC2987729

[ref15] Fan Y. C. Chiu H. C. Chen L. K. Chang G. J. J. Chiou S. S. (2015). Formalin inactivation of Japanese encephalitis virus vaccine alters the antigenicity and immunogenicity of a neutralization epitope in envelope protein domain III. PLoS Negl. Trop. Dis. 9:4167. doi: 10.1371/journal.pntd.0004167, 26495991 PMC4619746

[ref16] Feldmann F. Shupert W. L. Haddock E. Twardoski B. Feldmann H. (2019). Gamma irradiation as an effective method for inactivation of emerging viral pathogens. Am. J. Trop. Med. Hyg. 100, 1275–1277. doi: 10.4269/ajtmh.18-0937, 30860018 PMC6493948

[ref17] Fertey J. Bayer L. Grunwald T. Pohl A. Beckmann J. Gotzmann G. . (2016). Pathogens inactivated by low-energy-electron irradiation maintain antigenic properties and induce protective immune responses. Viruses 8:319. doi: 10.3390/v8110319, 27886076 PMC5127033

[ref18] Fertey J. Thoma M. Beckmann J. Bayer L. Finkensieper J. Reißhauer S. . (2020). Automated application of low energy electron irradiation enables inactivation of pathogen- and cell-containing liquids in biomedical research and production facilities. Sci. Rep. 10:12786. doi: 10.1038/s41598-020-69347-7, 32732876 PMC7393095

[ref19] Franka R. Wu X. Jackson F. R. Velasco-Villa A. Palmer D. P. Henderson H. . (2009). Rabies virus pathogenesis in relationship to intervention with inactivated and attenuated rabies vaccines. Vaccine 27, 7149–7155. doi: 10.1016/j.vaccine.2009.09.034, 19925945

[ref20] Furuya Y. Regner M. Lobigs M. Koskinen A. Müllbacher A. Alsharifi M. (2010). Effect of inactivation method on the cross-protective immunity induced by whole “killed” influenza a viruses and commercial vaccine preparations. J. Gen. Virol. 91, 1450–1460. doi: 10.1099/vir.0.018168-0, 20147516

[ref21] Gaidamakova E. K. Myles I. A. McDaniel D. P. Fowler C. J. Valdez P. A. Naik S. . (2012). Preserving immunogenicity of lethally irradiated viral and bacterial vaccine epitopes using a radio- protective Mn2+-peptide complex from Deinococcus. Cell Host Microbe 12, 117–124. doi: 10.1016/j.chom.2012.05.011, 22817993 PMC4073300

[ref22] Hankaniemi M. M. Stone V. M. Sioofy-Khojine A. B. Heinimäki S. Marjomäki V. Hyöty H. . (2019). A comparative study of the effect of UV and formalin inactivation on the stability and immunogenicity of a Coxsackievirus B1 vaccine. Vaccine 37, 5962–5971. doi: 10.1016/j.vaccine.2019.08.037, 31471148

[ref23] Hoffman E. A. Frey B. L. Smith L. M. Auble D. T. (2015). Formaldehyde crosslinking: a tool for the study of chromatin complexes. J. Biol. Chem. 290, 26404–26411. doi: 10.1074/jbc.R115.651679, 26354429 PMC4646298

[ref24] ISO 11137-2 (2013). European Committee for Standardization, Sterilization of Medical Products-Radiation. Part 2: Establishing the Sterilization Dose

[ref25] ISO/ASTM51607 (2013). Practice for use of the Alanine-EPR Dosimetry system, in Annual Book of ASTM Standards. ASTM International. Geneva, Switzerland: International Organization for Standardization, ISO.

[ref26] Jonah C. D. Spinks J. W. T. Woods R. J. (1990). Introduction to radiation chemistry. Radiat. Res. 124:378–379. doi: 10.2307/3577852

[ref27] Kelleher K. Subramaniam N. Drysdale S. B. (2025). The recent landscape of RSV vaccine research. Ther. Adv. Vaccines Immunother. 13:25151355241310601. doi: 10.1177/25151355241310601, 39802673 PMC11724408

[ref28] Lea D. E. (1946). The action of radiations on viruses and bacteria. Br. Med. Bull. 4, 24–26. doi: 10.1093/oxfordjournals.bmb.a072663, 21017183

[ref29] Lelie P. N. Reesink H. W. Lucas C. J. (1987). Inactivation of 12 viruses by heating steps applied during manufacture of a hepatitis B vaccine. J. Med. Virol. 23, 297–301. doi: 10.1002/jmv.1890230313, 2828525 PMC7166500

[ref30] Loveday E. K. Hain K. S. Kochetkova I. Hedges J. F. Robison A. Snyder D. T. (2021). Effect of inactivation methods on SARS-CoV-2 virion protein and structure. ACS Omega 6, 5624–5628. doi: 10.3390/v13040562 PMC8066162 33810401

[ref31] McEvoy B. Maksimovic A. Howell D. Reppert P. Ryan D. Rowan N. . (2023). Studies on the comparative effectiveness of X-rays, gamma rays and electron beams to inactivate microorganisms at different dose rates in industrial sterilization of medical devices. Radiat. Phys. Chem. 208:110915. doi: 10.1016/j.radphyschem.2023.110915

[ref32] Munir M. T. Federighi M. (2020). Control of foodborne biological hazards by ionizing radiations. Foods 9:878. doi: 10.3390/foods9070878, 32635407 PMC7404640

[ref33] Murphy B. R. Walsh E. E. (1988). Formalin-inactivated respiratory syncytial virus vaccine induces antibodies to the fusion glycoprotein that are deficient in fusion-inhibiting activity. J. Clin. Microbiol. 26, 1595–1597. doi: 10.1128/jcm.26.8.1595-1597.1988, 2459154 PMC266671

[ref34] Perera R. Pillai S. D. Alrubaye A. Jesudhasan P. (2025). Leveraging Electron beam (eBeam) Technology for Advancing the development of inactivated vaccines. Vaccines 13:179. doi: 10.3390/vaccines13020179, 40006726 PMC11861765

[ref35] Pollard E. (1953). Primary ionization as a test of molecular organization. Adv. Biol. Med. Phys. 3, 153–189. doi: 10.1016/b978-1-4831-9926-9.50008-2, 13030386

[ref36] Praveen C. Dancho B. A. Kingsley D. H. Calci K. R. Meade G. K. Mena K. D. . (2013). Susceptibility of murine norovirus and hepatitis a virus to electron beam irradiation in oysters and quantifying the reduction in potential infection risks. Appl. Environ. Microbiol. 79, 3796–3801. doi: 10.1128/AEM.00347-13, 23584781 PMC3675951

[ref37] Praveen C. McGrath I. A. Wavare N. S. Pillai S. D. (2025). Electron beam and X-ray Technologies in Agriculture and Food Processing: a viable alternative to Cobalt-60. Health Phys. 129, 160–173. doi: 10.1097/HP.0000000000002008, 40694634 PMC12637162

[ref38] Predmore A. Sanglay G. C. DiCaprio E. Li J. Uribe R. M. Lee K. (2015). Electron beam inactivation of Tulane virus on fresh produce, and mechanism of inactivation of human norovirus surrogates by electron beam irradiation. Int. J. Food Microbiol. 198, 28–36. doi: 10.1016/j.ijfoodmicro.2014.12.024, 25590261

[ref39] Reed L. J. Muench H. (1938). A simple method of estimating fifty per cent endpoints. Am. J. Epidemiol. 27, 192–197. doi: 10.1093/oxfordjournals.aje.a118408

[ref40] Salehi B. Motamedi-Sedeh F. Madadgar O. Khalili I. Langroudi A. G. C. Unger H. . (2018). Analysis of antigen conservation and inactivation of gamma-irradiated avian influenza virus subtype H9N2. Acta Microbiol. Immunol. Hung. 65, 163–171. doi: 10.1556/030.65.2018.025 29685054

[ref41] Schmidt T. Hoburg A. T. Gohs U. Schumann W. Sim-Brandenburg J. W. . (2012). Inactivation effect of standard and fractionated electron beam irradiation on enveloped and non-enveloped viruses in a tendon transplant model. Transfus. Med. Hemother. 39, 29–35. doi: 10.1159/000336380, 22896764 PMC3388620

[ref42] Schulze K. Weber U. Schuy C. Durante M. Guzmán C. A. (2024). Influenza virus inactivated by heavy ion beam irradiation stimulates antigen-specific immune responses. Pharmaceutics 16:465. doi: 10.3390/pharmaceutics16040465, 38675126 PMC11054185

[ref43] Shahrudin S. Chen C. David S. C. Singleton E. V. Davies J. Kirkwood C. D. . (2018). Gamma-irradiated rotavirus: a possible whole virus inactivated vaccine. PLoS One 13:e0198182. doi: 10.1371/journal.pone.0198182, 29879130 PMC5991763

[ref44] Singleton E. V. Gates C. J. David S. C. Hirst T. R. Davies J. B. Alsharifi M. (2021). Enhanced immunogenicity of a whole-inactivated influenza a virus vaccine using optimised irradiation conditions. Front. Immunol. 12:761632. doi: 10.3389/fimmu.2021.761632, 34899711 PMC8652198

[ref45] Sintara P. Suphaprueksapong P. Jetawattana S. Wiriyarat W. Akkhawattanangkul Y. Charngkaew K. . (2025). Safety and immunogenicity of inactivated Zika virus vaccine by gamma irradiation. Vaccine X 26:100706. doi: 10.1016/j.jvacx.2025.100706

[ref46] Skrobarczyk J. W. Martin C. L. Bhatia S. S. Pillai S. D. Berghman L. R. (2022). Electron-beam inactivation of human rotavirus (HRV) for the production of neutralizing egg yolk antibodies. Front. Immunol. 13:840077. doi: 10.3389/fimmu.2022.840077, 35359996 PMC8964080

[ref47] Suljovrujic E. Milicevic D. Stolic A. Dudic D. Vasalic D. Dzunuzovic E. . (2024). Thermal, mechanical, and dielectric properties of radiation sterilized mesomorphic PP: comparison between gamma and electron beam irradiation modalities. Polym. Degrad. Stab. 229:110940. doi: 10.1016/j.polymdegradstab.2024.110940

[ref48] Tallentire A. Miller A. (2015). Microbicidal effectiveness of X-rays used for sterilization purposes. Radiat. Phys. Chem. 107, 128–130. doi: 10.1016/j.radphyschem.2014.09.012

[ref49] Tallentire A. Miller A. Helt-Hansen J. (2010). A comparison of the microbicidal effectiveness of gamma rays and high and low energy electron radiations. Radiat. Phys. Chem. 79, 701–704. doi: 10.1016/j.radphyschem.2010.01.010

[ref50] Turan R. D. Tastan C. Dilek Kancagi D. Yurtsever B. Sir Karakus G. Ozer S. . (2021). Gamma-irradiated SARS-CoV-2 vaccine candidate, OZG-38.61.3, confers protection from SARS-CoV-2 challenge in human ACEII-transgenic mice. Sci. Rep. 11:15799. doi: 10.1038/s41598-021-95086-4, 34349145 PMC8339061

[ref51] Ward J. F. (1988). DNA damage produced by ionizing radiation in mammalian cells: identities, mechanisms of formation, and reparability. Prog. Nucleic Acid Res. Mol. Biol. 35, 95–125. doi: 10.1016/s0079-6603(08)60611-x, 3065826

[ref52] WHO (2011). Manual for the Laboratory Diagnosis and Virological Surveillance of influenza. Geneva, Switzerland: World Health Organization 2011.

[ref53] Wijewardana V. Ulbert S. Cattoli G. (2023). Editorial: irradiation technologies for vaccine development. Front. Immunol. 13:1075335. doi: 10.3389/fimmu.2022.1075335, 36700215 PMC9869150

[ref54] Wilton T. Dunn G. Eastwood D. Minor P. D. Martin J. (2014). Effect of formaldehyde inactivation on poliovirus. J. Virol. 88, 11955–11964. doi: 10.1128/jvi.01809-14, 25100844 PMC4178759

